# UASIS: Universal Automatic SNP Identification System

**DOI:** 10.1186/1471-2164-12-S3-S9

**Published:** 2011-11-30

**Authors:** Danny C C Poo, Shaojiang Cai, James T L Mah

**Affiliations:** 1 Department of Information Systems, School of Computing, National University of Singapore, 13 Computing Drive, Singapore 117417; 2 Data Mining Department, l2R, A*STAR, 1 Fusionopolis Way #21-01 Connexis (South Tower), Singapore 138632

## Abstract

**Background:**

SNP (Single Nucleotide Polymorphism), the most common genetic variations between human beings, is believed to be a promising way towards personalized medicine. As more and more research on SNPs are being conducted, non-standard nomenclatures may generate potential problems. The most serious issue is that researchers cannot perform cross referencing among different SNP databases. This will result in more resources and time required to track SNPs. It could be detrimental to the entire academic community.

**Results:**

UASIS (Universal Automated SNP Identification System) is a web-based server for SNP nomenclature standardization and translation at DNA level. Three utilities are available. They are UASIS Aligner, Universal SNP Name Generator and SNP Name Mapper. UASIS maps SNPs from different databases, including dbSNP, GWAS, HapMap and JSNP etc., into an uniform view efficiently using a proposed universal nomenclature and state-of-art alignment algorithms. UASIS is freely available at http://www.uasis.tk with no requirement of log-in.

**Conclusions:**

UASIS is a helpful platform for SNP cross referencing and tracking. By providing an informative, unique and unambiguous nomenclature, which utilizes unique position of a SNP, we aim to resolve the ambiguity of SNP nomenclatures currently practised. Our universal nomenclature is a good complement to mainstream SNP notations such as rs# and HGVS guidelines. UASIS acts as a bridge to connect heterogeneous representations of SNPs.

## Background

### Heterogeneous representations of SNPs

SNP, or Single Nucleotide Polymorphism, is defined as a bi-allele polymorphism at a single base with a frequency of more than 1% in the population [1,2]. Around 90% of the genome variations are limited to SNPs [3], which have been proven to be of great value for medical diagnostics and developing pharmaceutical products. They can also help identify multiple genes associated with complex diseases such as cancer and diabetes [4-6].

With the publication of the Human Genome Project (HGP) and emergence of next generation high-throughput sequencing techniques, there has been an explosion of data available for public use. SNP databases such as dbSNP [7], GWAS (formerly HGVbaseG2P) [8], HapMap [9] and JSNP [10] have collected millions of records. dbSNP, the largest one maintained by the National Center for Biotechnology Information (http://www.ncbi.nlm.nih.gov/SNP/), has collected 38,077,719 SNPs (rs#'s) for *Homo sapiens* to date (May 24, 2011, Build 132). The amount of data has been growing significantly. In addition, there are many more SNP databases, either public or private, that are used for pharmacogenetic research. An universal nomenclature is critical for clear, unequivocal and effective communication. However, it is widely recognized that heterogeneity of SNP nomenclatures and notations has complicated the process [3,11-14]. Table 1 lists the numerous alternative manners of designating a SNP in major databases. To make matter worse, private databases continue to use non-conventional representations that enlarge the set of possible nomenclatures as shown in Table 2[3].

**Table 1:** Alternative names of a SNP

Database	SNP names
dbSNP	rs3737965
	ss4923964, ss69366921
HGVBaseG2P	HGVM2256489
HGVS	NM_001286.2:c.87+45G>A, NM_021735.2:c.87+45G>A
	NM_021736.2:c.87+45G>A, NM_021737.2:c.87+45G>A
	NT_021937.19:g.7871183G>A
JSNP	IMS-JST083663
PharmGKB	rs3737965@chrl: 11789038
HapMap	rs3737965

**Table 2:** Non-conventional names of a SNP

dbSNP	rs28942082
Genome-browser-like syntax	Chr19:11,087,877-11,087,877 G/T
	Chr19:11087877 G/T
Others	geneA,11,EXON,108,T,hetero
	gene Asynonym,11,108,exon,GT
	proteinB, Gly564Val; proteinB, Bly544Val
	0014 FH NAPLES

There are many reasons for the existence of differing nomenclatures. Although Human Genome Variation Society (HGVS) has recommended widely-used guidelines for mutation notation, researchers of each laboratory have strong emotional attachment to their own naming system [15]. Research articles that first report novel SNPs do not always follow the HGVS guidelines, and the final genomic sequence is complied over many separate entries. Previous nomenclatures sometimes subsist for historical reasons. For example, *rs289↓2082* is still recorded as *"FH NAPLES"* or *"Bly544Val"* in OMIM (see Table 2).

### Problems of current SNP nomenclatures

Unambiguous and correct descriptions of SNPs in databases and in the literature are of utmost importance, not in the least since mistakes and uncertainties may lead to undesired errors in clinical diagnosis. HGVS nomenclature guidelines were proposed in as early as 1998 [16] then extended later on [17,18]. The guidelines have since been improved regularly (http://www.hgvs.org/mutnomen/). However, the sole existence of the guidelines by themselves is not sufficient. The standardization of SNP identification is far from complete [11,12,14].

It is clear that dbSNP is becoming a major center for deposition of SNPs from various sources. The SNP nomenclature of dbSNP, rs#, is unique, clear and stable. It has been widely adopted and heavily referenced in the literature. JSNP, GWAS, HapMap and PharmGKB provide corresponding rs# when displaying their own records. We highly respect its authority.

It is noted that overlapping of SNPs is very low (around 1%) among recognized databases [12]. JSNP reported only 20.9% identity compared to dbSNP [10]. Researchers have to submit their SNPs to dbSNP before they can get a rs#. However, some SNPs discovered in the research or diagnostic laboratory may even never be reported in any publication or database. Some SNPs have considerable delays in their public release due to commercial agreements, legal considerations or ethical reasons [6,19]. They are unlikely to be assigned identifiers that can be uniformly used later on. Even for dbSNP itself, there are many rs#'s abandoned due to regular clustering [20]. These identifiers may have been cited in publications, leading to confusion and ambiguity.

Another candidate is HGVS mutation nomenclature guidelines, which are largely adopted by researchers and enforced by some journals. The format is like *"*<*Accession Number*>.<*version number*>(<*Gene symbol*>)*:*<*sequence type*>.<*mutation*>*"*. However, it is not universally applied as a standard, since it is complex and not unique. Table 1 gives five alternative names that are legal for a SNP, where the coordinate systems are based on different reference sequences. The mutation position is obtained based on some reference sequences. In addition, reference sequences are evolving with each new version. That makes the names unstable. More effort is thus required to translate data in published papers and databases between different versions of reference sequences [21,22]. Finally, the names may be too long and complex to remember and communicate.

Current SNP nomenclatures, including rs#, are mostly arbitrary combination of letters and digits maintained by manual curation. The major problem is that they are not informative and only available within a single database. Automatic ways of mapping SNPs based on their names are rare. One way is to perform searching in available databases separately, and then compare the obtained records manually. For example, given only SNP names, we are unable to answer these kind of simple questions: *What SNPs have been discovered on gene CHR1* (*chromosome 5*, *locus 26648951..26653073*)*?* or *What diseases have been found closely associated to rs28942082?* HGVS nomenclature is searchable and informative, but suffers from complexity and non-unique feature.

With different nomenclatures, it is difficult to cross reference SNPs among the various databases. Research based on the data only from one SNP database will lead to an incomplete compilation of variants and inadequate genomic analysis. For researchers who track SNPs through literature scanning, it is very difficult to gain a global picture from overwhelming publications since SNPs are not uniformly searchable in the literature. It is also not possible to search by position or polymorphism information. That could be a tough data mining challenge, which consumes considerable resources and time. From the discussion above, we believe that the existing SNP nomenclatures do not provide a universal standard.

### SNP standardization and database integration

Tremendous efforts have been made to keep SNP data uniformly. Besides the continuous development of HGVS nomenclature guidelines, SNP databases are integrating data from more sources.

GWAS, previously HGVbaseG2P, is one of the largest SNP databases [23,24]. It gathers information of SNPs from the literature, their own and collaborative discovery efforts and unsolicited submissions. It exchanges core data with dbSNP regularly. The pharmacogenomics knowledge base (PharmGKB) allows cross-referencing against dbSNP, JSNP and HapMap, as well as other sources such as UCSC Genome Browser [25].

Some applications focus on retrieving SNPs fulfilling certain criteria such as locus and haplotype tagging. SNPper is web-based platform to search and export SNP records from dbSNP [26]. TAMAL (Technology And Money Are Limiting) provides a query portal to latest versions of five SNP sources (HapMap, Perlegen, Affymetrix, dbSNP and the UCSC genome browser) [27]. It helps to select SNPs that are likely involved in the genetic determination of human complex traits. LS-SNP annotates from dbSNP the coding of non-synonymous SNPs (nsSNPs) that will result in mutation in protein [28]. Other works place emphasis on intragenic SNPs [29].

Among the previous works carried out, Mutalyzer sequence variation nomenclature checker [14] and SNP-Converter [3] are similar to the work described here. These two applications aim to support HGVS nomenclature guidelines. Mutalyzer checks if an SNP name follows the HGVS guidelines. Furthermore, it is capable of generating legal identifiers given the pivot features of a SNP. SNP-Converter converts whatever SNP names into HGVS names by exploring certain gene databases to determine the correct locus. It treats the integration process as a knowledge mining task. SNP-Converter is based on a complete SNP notation in XML format, acting as an ontology, to create a uniform semantic environment [3,30].

## Implementation: universal SNP nomenclature and UASIS

From the discussions above, it is clear that dbSNP is an important database that cannot be ignored by any application. However, it does take considerable effort to translate nomenclatures among the SNP databases. To overcome the shortcomings of rs# and HGVS nomenclatures, we propose a universal nomenclature and UASIS (Universal Automated SNP Identification System). We believe our nomenclature is a good complement to rs# and HGVS, acting as a bridge connecting various databases, including private and unpublished ones. A system of nomenclature has to strike a compromise between the convenience and simplicity required for everyday use and the need for adequate definition of the concepts involved [31]. In 2006, Human Variome Project Meeting gathered leading representatives to discuss key problems of human gene variation industry [13]. The meeting gave 96 recommendations. Two of them regarding to "Nomenclatures and Standards" are:

*4. Develop tools to accurately translate and search earlier nomenclature systems into successor systems*.

*6. The most current genome build be unambiguously adapted as the reference sequence*, *and that a standard be developed for the submission of all variant data that includes both a genome coordinate as well as sufficient flanking sequence to map the variation independently*.

UASIS is inspired from these two requirements. UASIS proposes a universal nomenclature for SNPs with the form *"*<*human genome version*> . <*chromosome number*>*:*<*locus*>*:*<*alleles*>*"*. Detailed specification is shown in Table 3. According to this specification, SNP *rs3737965* is represented as *HG19.1:11789038:G/A*, indicating a pair of alleles *"G"* and *"A"* at position 11789038 of chromosome 1, and the position is based on human reference genome version 19. Note that for indels, the polymorphism occurs *at* the position given. For example, *"1234insT"* means that *"T"* is placed at position 1234, and the original one, say, *"C"* is at position 1235.

**Table 3:** Universal SIMP nomenclature

Syntax	Example	Description
HG( *numeric version*)	HG19	Complete human reference genome
		by UCSC. '19' is version number
Chr number	1..22, X, Y	Chromosome numbers
Numeric	21898363	1-based position
Nucleotides	**A**, **C**, **G**, **T**, **N**	**N** for unclear nucleotide
/	G/A	Substitution: alleles are 'G' and 'A'
ins	insA	Insertion: 'A' is inserted
del	delT	Deletion: 'T' is deleted

Compared to HGVS guidelines, we fix the coordinate to be the whole human genome. And we give only one position without "_", since we consider only single bi-allele mutations. The first advantage is that it allows for succinct comparison using the accession numbers. The nomenclature is based on the human reference genome and not any *arbitrary* reference sequences, resulting in the generation of unique identifiers. All SNPs would be given the same prefix *"HG19"* currently. Secondly, it is unambiguous, informative and stable since the name consists of all necessary information to uniquely define an SNP. More importantly, UASIS nomenclature gives names that are searchable and comparable. It helps SNP tracking in the literature if universally adopted.

Another difference is the representation of mutations. HGVS guidelines use a ">" symbol to mean " changed to". Here we only list all possible alleles delimited by a " /". *"A/T"* means that the major allele could be either *"A"* or *"T"*. Normally the first is the one on the reference genome. This definition is for simplicity. Determining the frequency of alleles requires more effort in the laboratory. In different populations or laboratory testings the results could be non-identical. For SNPs which have more than two alleles, the " >" symbol will lose its clarity, leading to ambiguity. This syntax is also used by other browser viewers [3]. But we would recommend that the leftmost allele should be the major allele.

The most important advantage of UASIS nomenclature is that, unlike rs#, it does not depend on any particular database. The naming process of an SNP can be done automatically, regardless of the database maintaining it, or the contig the SNP is derived from, etc. Researchers do not necessarily submit to a particular database to get identifiers. They will get names instantaneously without waiting for manual approval using UASIS. Although dbSNP designates a ss# once a SNP is submitted, the ss# suffers similar problems of rs#. For private SNPs that cannot be published due to various reasons, UASIS nomenclature is obviously a better choice.

UASIS nomenclature is not intended to replace the rs# since rs# already has significant influence on SNP nomenclatures, rs#'s are simple, unique and stable. Actually, UASIS nomenclature is a good complementary to rs#, playing a similar role as ss#. But we believe that it is more than ss# and it will benefit the whole process of SNP standardization. One disadvantage of our notation is that it depends on the human reference genome. That is an unavoidable trade off given all attractive benefits of our universal nomenclature. But HG19 is considered as *"finished"* by the Genome Reference Consortium. We expect a much lower updating frequency of human genome in future.

UASIS is a web-based server system (http://www.uasis.tk) for annotating novel SNPs and cross-referencing among databases instantaneously. There are utility tools available, i.e., UASIS Aligner and Universal SNP Name Generator. For newly discovered SNPs, UASIS aligner performs efficient sequence alignment and checks whether the polymorphism has been deposited in main databases, including GWAS, dbSNP, JSNP and HapMap. In addition, for each mutation, UASIS provides an identifier based on our proposed nomenclature as described above. These identifiers can be used immediately and instantaneously. In this way, researchers are free to map SNPs among various nomenclatures. More databases like PharmGKB are currently in the process of being integrated into UASIS. Universal SNP Name Generator and SNP Name Mapper take in information of a SNP and perform cross-checking among main databases.

UASIS is available at http://www.uasis.tk since August 2010. It is implemented in PHP and MySQL, and designed for various types of web browser. Detailed information on the use of UASIS is provided online at the website.

### UASIS Aligner

#### Input

**Figure 1:**
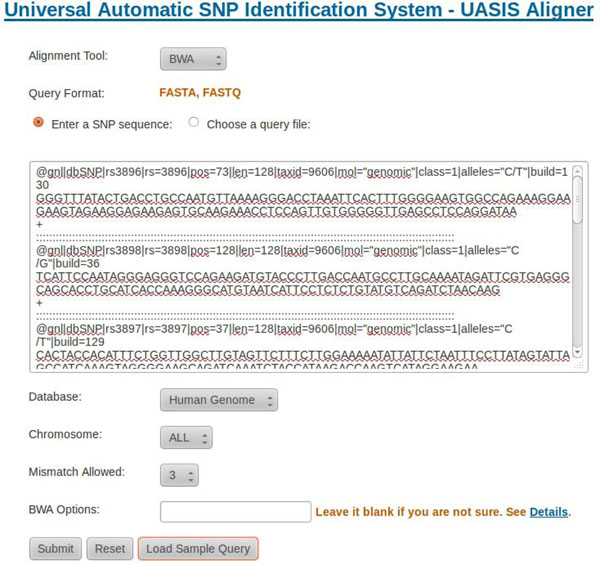
**Input of UASIS Aligner**. Users could choose to upload the flanking sequences of SNPs as file, or input the sequences directly. Currently we support FASTQ format for Bowtie and FASTA/FASTQ for BWA. Other parameters include which chromosome to align and how many mismatches allowed.

Users upload flanking sequences of SNPs explicitly or by uploading a file in FASTQ or FASTA format. They could choose underlying alignment tool, which chromosome to align, and how many mismatches allowed according to query characteristics. Meanwhile, they are able to specify advanced options manually. The human reference genome used is based on HG19, downloaded from UCSC (http://genome.ucsc.edu/). Figure 1 showes the screenshot using the sample data.

#### Sequence alignment

Efficiency and accuracy are critical for real time systems like UASIS. Bowtie [32] and BWA [33] are winners [20]. Mah et al. conducted rigorous experiments to compare popular alignment tools MAQ, SOAP2, BWA and Bowtie with BLAST results as the benchmark. The results proved that MAQ could not handle reads longer than 76bp and SOAP2 was memory inefficient. Bowtie and BWA are able to align thousands of sequences every second. Both tools are developed based on Burrows-Wheeler Transform (BWT) [34] data structure and FM-index [35]. Bowtie is optimized for short reads around 35 base pair, which is the output read length of NGS (Next Generation Sequencing) platforms Illumina Solexa and SOLiD [36]. It supports up to 3 mismatches by enumerating all possible permutations. This strategy makes it ultra fast, but it does not support gapped alignment. BWA employs roughly the same idea but it implements gapped alignment.

NGS techniques are producing longer and longer reads, for instance, 454 (around 400bp) and Illumina (a few hundreds base pairs). Bowtie and BWA are sufficient to perform long read alignments if there are just a few mismatches. Bowtie supports queries up to 1024bp [32]. Mah et al. have shown that BWA and Bowtie achieved high accuracy and efficiency for reads up to 1024bp [20]. UASIS works perfectly for NGS sequences. In this study, we conducted experiments based on reads up to 512bp. For reads longer than 1024bp, we would explore some specific alignment tools in future, such as BWA-SW [37].

Query sequences are uploaded and aligned to reference human genome by executing Bowtie or BWA. Then UASIS checks whether the query SNP exists in dbSNP, GWAS, JSNP or HapMap by inspecting the allele position. UASIS is very responsive since the alignment tools are efficient.

#### Output

**Figure 2:**
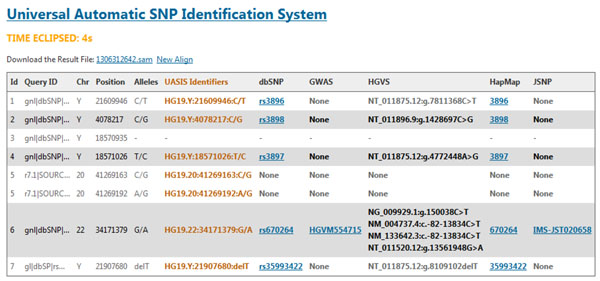
**Result of UASIS Aligner**. Align the flanking sequences of SNPs submited by users. The alignment is performed by Bowtie or BWA. If the SNP is found, search it in databases dbSNP, JSNP, GWAS and HapMap.

Alignments will be listed in tabular form, including query id, allele position, alleles, UASIS identifier, dbSNP rs#, GWAS id, JSNP id, HapMap id. Given the polymorphism position, we are able to obtain corresponding identifiers recorded in dbSNP, JSNP and HapMap. If no record is found in a database, a *"none"* message will be displayed for that database. Results in SAM format can be downloaded for further analysis. Figure 2 illustrates the sample output of UASIS Aligner.

#### Experiments

To evaluate the accuracy and efficiency of UASIS, we conducted experiments on simulated and real SNPs with length 35, 76, 128 and 512bp, and performed cross-checking between dbSNP and JSNP. CPU time on a quad core of a 2.4 GHz Xeon E5620 processor with 16G RAM and accuracy in percentage are evaluated (see Table 4).

**Table 4:** Alignment evaluation on simulated & real SNPs

	Simulated SNPs			Real SNPs		
Program	Reads	Time (s)	Accuracy (%)	Reads	Time (s)	Accuracy (%)

Bowtie-35	24326	2	76.4	72241	4	90.5
BWA-35	24326	5	83.4	72241	8	90.2
Bowtie-76	24359	3	89.2	72241	4	93.4
BWA-76	24359	9	92.8	72241	19	92.8
Bowtie-128	24373	2	90.1	72241	5	92.4
BWA-128	24373	5	94.7	72241	49	93.4
Bowtie-512	24373	3	93.8	72241	9	93.5
BWA-512	24373	23	97.2	72241	132	91.7

Totally 94771 reads of 35, 76, 128 and 512bp were simulated from human genome build 37.1 with error rates following Sanger sequencing pattern. Such that for each read, average amount of mismatches and indels is 1 and 0.02. CPU time on a quad core of a 2.4 GHz Xeon E5620 processor with 8G RAM and accuracy in percentage are given.

94771 reads were simulated from the human genome (Build 37.1) using MetaSim [38] package following the error pattern of Sanger reads. Meanwhile, 72241 flanking sequences were downloaded from dbSNP (ftp://ftp.ncbi.nih.gov/snp/organisms/human_9606/rs_fasta/) and JSNP (http://snp.ims.u-tokyo.ac.jp/map/Dump/). For Bowtie, we use the options "-best -k 2 -v 3", meaning that it will report at most two hits allowing three mismatches in decreasing quality order. And for BWA, the options are "-n 3 -o 3", meaning that the edit distance is at most three and there are at most three gaps.

For both dataset, all three tools were found to show reliability. As the read length grows, the accuracy improves. Bowtie generated higher error rate since it does not support gapped alignment. But Bowtie was very efficient, taking less than 4 seconds to process.

UASIS is also introduced briefly on CBAS-SYMBIO 2010 held in Singapore. Approximately 30 people outside UASIS group have tested it.

### Universal SNP Name Generator

**Figure 3:**
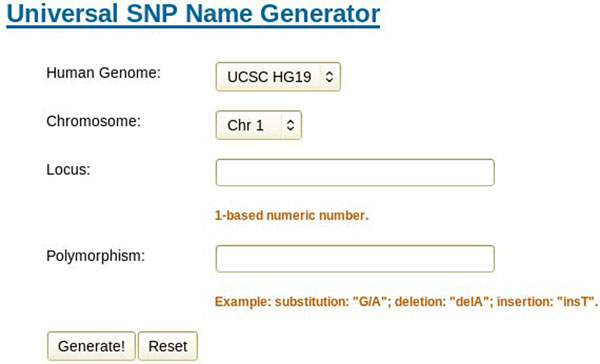
**Input of Universal SNP Name Generator.** Show the input options of Universal SNP Name Generator. Users are supposed to provide human genome version, chromosome number, locus and alleles.

**Figure 4:**
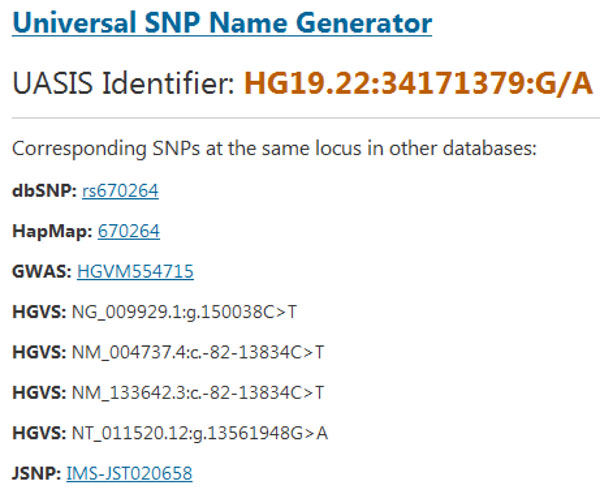
**Result of Universal SNP Name Generator.** Generate UASIS identifier given the pivot features. If there are records deposited in existing databases, show corresponding identifiers and links.

Similar to Mutalyzer [14], our generator takes in all pivot features that define a SNP uniquely. The features include reference genome, chromosome, position and alleles. Please note that for the mutation position of SNPs, different databases use different coordinate. dbSNP, the largest public one, uses 1-based positions. However, in the dump database files, the position is 0-based. And JSNP uses 1-bases positions in its dump database file. Here we choose 1-based strategy for consistency. The generator performs validation strictly to ensure the user input is legal. Figure 3 is a screenshot of the input page. But instead of HGVS names, we generate our UASIS identifiers as the result, as well as corresponding HGVS names and access ids in dbSNP, GWAS, HapMap and JSNP. Currently GWAS is not providing downloadable SNP files, so we utilize the online query system of GWAS with rs# as the keyword. HGVS and JSNP identifiers are obtained from local databases recording relationship between them. When performing the cross-referencing, we only check whether there is a SNP at the same locus, regardless the alleles. But it is now sufficient for researchers. More functionality is under development. Figure 4 is the output of sample data.

### SNP Name Mapper

**Figure 5:**
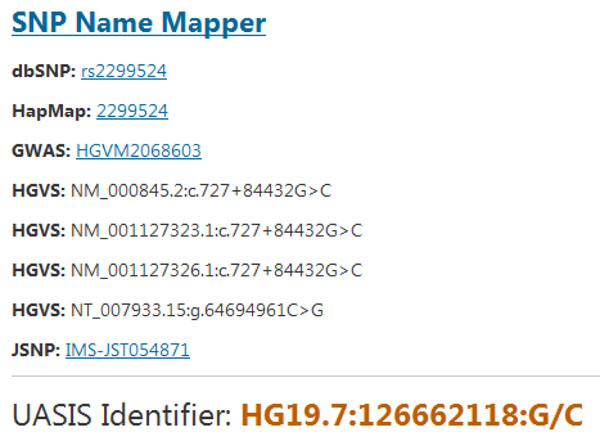
**Result of SNP Name Mapper**. Generate UASIS identifier given a particular SNP name. If there are records deposited in existing databases, show corresponding identifiers and links.

The SNP Name Mapper performs similar task to Name Generator. However, it is more suitable for researchers who have some SNPs at hand, and would like to know what related works have been done in the literature. Users are required to provide an existing SNP name from certain database. For example, *"rs3897"* from dbSNP. If the input name is not valid, a *"None"* message will be displayed. Figure 5 illustrates a sample output of this utility. We also generate corresponding identifier following our universal nomenclature (see Section Implementation). The alleles information can only be obtained from two sources. If a JSNP record exists, there are alleles deposited. Otherwise, we search the online query system of dbSNP and parse the result page to extract the alleles information. If no rs# is available, we would not generate UASIS identifier.

## Conclusions

Differing SNP nomenclatures have been a large concern for a long period. UASIS (Universal Automated SNP Identification System) proposes an informative, unique and unambiguous nomenclature that serves as a good complement to the present methods of identifying SNPs. The universal nomenclature is important for naming newly discovered or unpublished SNPs. The most significant advantage is that it provides a bridge to cross reference SNP identifiers among various databases. UASIS Aligner is an utility to perform pairwise sequence alignment and cross referencing in real time (<20s). Through Universal Name Generator and SNP Name Mapper, SNPs from dbSNP, GWAS, JSNP and HapMap can be mapped to one another. More databases are being integrated into UASIS. UASIS not only helps to achieve uniform notation of SNPs in the literature, but also aid in determining accurate SNP genotypes and haplotypes.

## Availability and requirements

Project name: UASIS (Universal Automated SNP Identification System)

Project home page: http://www.uasis.tk with no requirement of log-in.

Hardware specifications: Dell T710, quad core of a 2.4 GHz Xeon E5620 processor, 8G RAM

Operating system(s): Ubuntu Server 10.04, kernel 2.6.32

Programming language: C++ and PHP web interface, could be assessed by various browsers, including Molliza Firefox, Chrome, Internet Explorer, etc

Database: MYSQL 5.1.41, storing 68382797 SNP records from dbSNP and JSNP

## Limitations

UASIS focuses on the standardization of single nucleotide polymorphisms. Currently we do not handle more complicated variations, including reversions, deletion/insertions of multiple bases, rearrangements and CNVs (Copy Number Variations) [39]. Because the nomenclatures of these variations require much more effort to reach a consensus. To the best of our knowledge, we have not found any efficient approaches to discover these variations instantaneously. We would leave this exciting topic in future studies. Users are able to perform batch processing through UASIS Aligner with constraints. The maximum upload size is 5MB. And only files with extensions fa, fas, fast, fasta, fq and fastq are allowed. If the query format is incorrect, UASIS Aligner will report an error message or list the results as "Not Aligned". A third limitation is the synchronization between UASIS and SNP databases. Now we store the relationship of nomenclatures from dbSNP, JSNP, HGVS and HapMap as a local repository. Once these databases update the records, we have to update our local copy manually. For GWAS, we fetch the webpage through its online query system and then extract necessary information. In this case, if the query system is changed, we should change the code accordingly. From our observations, all of these databases have not performed major changes in the past half a year. We believe that UASIS is relatively stable.

## Competing interests

The authors declare that they have no competing interests.

## Authors contributions

Dr. Poo and Dr. Mah proposed the novel idea of universal nomenclature, reviewed and compared various SNP nomenclatures. Mr. Cai completed the universal nomenclature, developed the web server system, conducted the experiments and drafted this paper.

## References

[B1] Brookes A The essence of SNPs Gene 1999 234 2 177 186 10.1016/S0378-1119(99)00219-X 10395891

[B2] Su SC Jay Kuo CC Chen T Single nucleotide polymorphism data analysis - state-of-the-art review on this emerging field from a signal processing viewpoint Signal Processing Magazine, IEEE 2007 24 75 82

[B3] Coulet A Smaϊl-Tabbone M Benlian P Napoli A Devignes MD SNP-Converter: an ontology-based solution to reconcile heterogeneous SNP descriptions for pharmacogenomic studies Data Integration in the Life Sciences 2006 4075 82 93 10.1007/11799511_8

[B4] Miller RD Kwok PY The birth and death of human single-nucleotide polymorphisms: new experimental evidence and implications for human history and medicine Human Molecular Genetics 2001 10 20 2195 2198 10.1093/hmg/10.20.2195 11673401

[B5] Tamura K Suzuki M Arakawa H Tokuyama K Morikawa A Linkage and association studies of STAT6 gene polymorphisms and allergic diseases International Archives of Allergy and Immunology 2003 131 33 38 10.1159/000070432 12759487

[B6] Horaitis O Cotton RG The challenge of documenting mutation across the genome: the human genome variation society approach Hum Mutat 2004 23 5 447 452 10.1002/humu.20038 15108276

[B7] Smigielski EM Sirotkin K Ward M Sherry ST dbSNP: a database of single nucleotide polymorphisms Nucl. Acids Res 2000 28 352 355 10.1093/nar/28.1.352 10592272 PMC102496

[B8] Brookes AJ Lehvaslaiho H Siegfried M Boehm JG Yuan YP Sarkar CM Bork P Ortigao F HGBASE: a database of SNPs and other variations in and around human genes Nucleic Acids Res 2000 28 356 360 10.1093/nar/28.1.356 10592273 PMC102467

[B9] The international HapMap project Nature 2003 426 6968 789 796 10.1038/nature02168 14685227

[B10] Hirakawa M Tanaka T Hashimoto Y Kuroda M Takagi T Nakamura Y JSNP: a database of common gene variations in the Japanese population Nucleic Acids Res 2002 30 158 162 10.1093/nar/30.1.158 11752280 PMC99126

[B11] den Dunnen JT MH P Standardizing mutation nomenclature: why bother? Hum Mutat 2003 22 3 181 182 10.1002/humu.10262 12938082

[B12] Marsh S Kwok P McLeod HL SNP databases and pharmacogenetics: great start, but a long way to go Human Mutation 2002 20 3 174 179 10.1002/humu.10115 12203989

[B13] Cotton RGH Recommendations of the 2006 Human Variome Project meeting Nat Genet 2007 39 4 433 436 10.1038/ng2024 17392799

[B14] Wildeman M van Ophuizen E den Dunnen JT Taschner PE Improving sequence variant descriptions in mutation databases and literature using the Mutalyzer sequence variation nomenclature checker Human Mutation 2008 29 6 13 10.1002/humu.20654 18000842

[B15] Wain H White J Povey S The changing challenges of nomenclature Cytogenet Cell Genet 1999 86 2 162 164 10.1159/000015372 10545710

[B16] SE A the Nomenclature Working Group Recommendations for a nomenclature system for human gene mutations Hum Mutat 1998 11 1 3 9450896 10.1002/(SICI)1098-1004(1998)11:1<1::AID-HUMU1>3.0.CO;2-O

[B17] den Dunnen JT Antonarakis SE Mutation nomenclature extensions and suggestions to describe complex mutations: a discussion Human mutation 2000 15 7 12 10.1002/(SICI)1098-1004(200001)15:1<7::AID-HUMU4>3.0.CO;2-N 10612815

[B18] den Dunnen JT Antonarakis SE Nomenclature for the description of human sequence variations Human genetics 2001 109 121 124 10.1007/s004390100505 11479744

[B19] Cotton RGH Horaitis O Quality control in the discovery, reporting, and recording of genomic variation Human Mutation 2000 15 16 21 10.1002/(SICI)1098-1004(200001)15:1<16::AID-HUMU6>3.0.CO;2-S 10612817

[B20] Mah JT Poo DC Cai S UASMAs (Universal Automated SNP Mapping Algorithms): a set of algorithms to instantaneously map SNPs in real time to aid functional SNP discovery Proc. VLDB2010 Endow 2010 3 1-2

[B21] Dalgleish R Flicek P Cunningham F Astashyn A Tully R Proctor G Chen Y McLaren W Larsson P Vaughan B Beroud C Dobson G Lehvaslaiho H Taschner P den Dunnen J Devereau A Birney E Brookes A Maglott D Locus Reference Genomic sequences: an improved basis for describing human DNA variants Genome Medicine 2010 2 4 24 10.1186/gm145 20398331 PMC2873802

[B22] Fokkema IFAC Taschner PEM Schaafsma GCP Celli J Laros JFJ den Dunnen JT LOVD v.2.0: the next generation in gene variant databases Human Mutation 2011 32 5 557 563 10.1002/humu.21438 21520333

[B23] Fredman D Munns G Rios D Sjöholm F Siegfried M Lenhard B Lehväslaiho H Brookes AJ HGVbase: a curated resource describing human DNA variation and phenotype relationships Nucleic Acids Research 2004 32 suppl 1 D516 D519 14681471 10.1093/nar/gkh111 PMC308845

[B24] Thorisson GA Lancaster O Free RC Hastings RK Sarmah P Dash D Brahmachari SK Brookes AJ HGVbaseG2P: a central genetic association database Nucleic Acids Research 2009 37 suppl 1 D797 D802 18948288 10.1093/nar/gkn748 PMC2686551

[B25] Hewett M Oliver DE Rubin DL Easton KL Stuart JM Altman RB Klein TE PharmGKB: the Pharmacogenetics Knowledge Base Nucl. Acids Res 2002 30 163 165 10.1093/nar/30.1.163 11752281 PMC99138

[B26] Riva A Kohane IS SNPper: retrieval and analysis of human SNPs Bioinformatics 2002 18 12 1681 1685 10.1093/bioinformatics/18.12.1681 12490454

[B27] Hemminger BM Saelim B Sullivan PF TAMAL: an integrated approach to choosing SNPs for genetic studies of human complex traits Bioinformatics 2006 22 5 626 627 10.1093/bioinformatics/btk025 16418238

[B28] Karchin R Diekhans M Kelly L Thomas DJ Pieper U Eswar N Haussler D Sali A LS-SNP: large-scale annotation of coding non-synonymous SNPs based on multiple information sources Bioinformatics 2005 21 12 2814 2820 10.1093/bioinformatics/bti442 15827081

[B29] Aerts J Wetzels Y Cohen N Aerssens J Data mining of public SNP databases for the selection of intragenic SNPs Human Mutation 2002 20 3 162 173 10.1002/humu.10107 12203988

[B30] Coulet A Smaϊl M Tabbone Benlian P Napoli A Devignes MD SNP-Ontology for semantic integration of genomic variation data, 14th Annual International Conference on Intelligent Systems for Molecular-Biology - ISMB'06 200617274837

[B31] Bodmer WF HLA: what's in a name? Tissue Antigens 1997 49 3 293 296 10.1111/j.1399-0039.1997.tb02758.x 9098944

[B32] Langmead B Trapnell C Pop M Salzberg SL Ultrafast and memory-efficient alignment of short DNA sequences to the human genome Genome biology 2009 10 3 R25+ 19261174 10.1186/gb-2009-10-3-r25 PMC2690996

[B33] Li H Durbin R Fast and accurate short read alignment with Burrows-Wheeler transform Bioinformatics 2009 25 14 1754 1760 10.1093/bioinformatics/btp324 19451168 PMC2705234

[B34] Burrows M Wheeler DJ A block-sorting lossless data compression algorithm Tech. Rep. 124 1994 Digital Equipment Corporation

[B35] Ferragina P Manzini G Opportunistic data structures with applications Foundations of Computer-Science, Annual IEEE Symposium 2000 0 390 398

[B36] Wall PK Leebens-Mack J Chanderbali A Barakat A Wolcott E Liang H Landherr L Tomsho L Hu Y Carlson J Ma H Schuster S Soltis D Soltis P Altman N dePamphilis C Comparison of next generation sequencing technologies for transcriptome characterization BMC Genomics 2009 10 347 10.1186/1471-2164-10-347 19646272 PMC2907694

[B37] Li H Durbin R Fast and accurate long-read alignment with Burrows-Wheeler transform Bioinformatics (Oxford, England) 2010 26 5 589 595 10.1093/bioinformatics/btp698 PMC2828108 20080505

[B38] Richter DC Ott F Auch AF Schmid R Huson DH MetaSim—A Sequencing Simulator for Genomics and Metagenomics PLoS ONE 2008 3 10 e3373 10.1371/journal.pone.0003373 18841204 PMC2556396

[B39] Tuzun E Sharp AJ Bailey JA Kaul R Morrison VA Pertz LM Haugen E Hayden H Albertson D Pinkel D Olson MV Eichler EE Fine-scale structural variation of the human genome Nature Genetics 2005 37 7 727 732 10.1038/ng1562 15895083

